# Local Genetic Adaptations Among Remnant Populations of British Common Juniper, Juniperus communis, Indicated by a Common Garden Trial

**DOI:** 10.1002/ece3.71049

**Published:** 2025-03-17

**Authors:** J. Baker, J. Cottrell, R. Ennos, A. Perry, S. Green, S. Cavers

**Affiliations:** ^1^ UKCEH Penicuik UK; ^2^ University of Edinburgh Edinburgh Scotland; ^3^ Forest Research and University of Edinburgh Edinburgh Scotland

**Keywords:** common garden trial, genetic diversity, genetic isolation, habitat fragmentation, juniper, local adaptations, quantitative genetics

## Abstract

Habitat fragmentation and genetic isolation pose threats to the genetic diversity and resilience of natural populations. Protecting the genetic diversity of populations, and the processes that sustain it, optimizes their ability to adapt to changing conditions and new threats: an approach known as “dynamic conservation.” The common juniper, *Juniperus communis* , is a keystone species that provides habitat and resources for many plants and animals. It is highly polymorphic, and across its natural range is found in a variety of habitats and diverse growth forms. Juniper populations have been shrinking and becoming increasingly fragmented for over a century in the UK and elsewhere in Europe, raising concerns about the genetic diversity present in remnant populations and their capacity to adapt to changing conditions or adaptive potential. This paper presents an analysis of the partitioning of phenotypic diversity among regions, populations, and families from 16 UK populations assessed in a common garden trial. Our findings suggest high phenotypic variation among populations compared to the variation among families within populations, indicating barriers to gene flow between juniper populations, relatively homogeneous populations, and therefore potentially reduced adaptive potential. This information is a useful baseline for conservation managers and will help to protect the genetic diversity and adaptive potential of populations.

## Graphical abstract

The quantitative genetics of UK populations of juniper were evaluated. We found evidence that juniper is less capable of gene flow than other species of conifers.

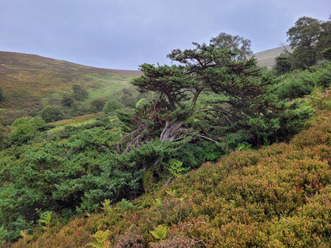

## 1 Introduction

The capacity of species to adapt to changing conditions is underpinned by genetic variation and the extent to which it is heritable across generations (Boyd et al. 2022; Geng et al. 2016). Within a species, genetic diversity is partitioned among populations according to the extent of gene flow, migration, and selection operating within and among those populations. Maintaining genetic diversity by ensuring a dynamic balance between gene flow and natural selection can support adaptive potential and therefore resilience. Strategies that aim to do so are typically termed “dynamic conservation” (Cavers and Cottrell 2015; Eriksson et al. 1993), and they have been adopted as a genetic resource conservation approach, particularly for tree species (Lefèvre et al. 2013). Here, we aim to evaluate the genetic variation and adaptive potential in UK populations of common juniper, *Juniperus communis*, using a common garden trial. Throughout, we refer to geographically discrete stands of trees as “populations,” which in the case of UK juniper are typically small and fragmented. Juniper is one of only three conifers native to the UK, and despite intensive conservation efforts in some areas such as in Southern England, populations are shrinking and becoming increasingly fragmented. Some stands have declined to the point of senescence and have failed to reproduce naturally in recent years. This decline may have serious repercussions for the genetic diversity, and therefore adaptive potential and resilience, of the UK's remaining juniper, especially given the changing climate and the introduction of non‐native pathogens. We quantified phenotypic diversity in 16 juniper populations to gain an understanding of the standing genetic variation, or the amount of “raw [genetic] material” (Aguilar et al. 2008) upon which natural selection can act. As adaptive potential depends on phenotypic variation (changing conditions will impose selection, and selection acts on the phenotype), the most valuable assessment of genetic diversity is to quantify the genetic component of phenotypic variation. This information can help conservation managers understand how juniper may tolerate both its current and future stressors.

Common garden trials are used by researchers to assess the genetic variation underpinning phenotypic traits (de Villemereuil et al. 2016; Ramírez‐Valiente et al. 2021; Schwinning et al. 2022). Since a phenotype is a product of both genes and the environment, common garden trials allow quantification of the genetic part of phenotypic trait variation by minimizing the effects of the environment. This information can be used to identify locally adaptive traits (McKay et al. 2001) and guide conservation efforts, including informing the debate about the need to transplant individuals between sites in such a way as to minimize the risks of maladaptation (Schwinning et al. 2022). Furthermore, if the trial design incorporates pedigree information (such as family identity), heritability can be estimated to elucidate the patterns of local adaptation that selection may act upon (de Villemereuil et al. 2016; Donnelly et al. 2016; Schwinning et al. 2022). Finally, patterns of variation in putatively adaptive traits identified in common garden trials can be correlated with local climate variables from the source sites to improve our understanding of the climatic drivers of local adaptation (López et al. 2008; McKay et al. 2001; Ramírez‐Valiente et al. 2021; Schwinning et al. 2022). This paper evaluates genetically based phenotypic diversity both within and among progeny from 16 natural UK juniper populations using a common garden trial to provide a genetic baseline assessment of standing phenotypic genetic diversity in this UK Biodiversity Action Plan priority species.

The common juniper is the most widespread conifer in the world, with a circumpolar range spanning from the tundras of Russia and Canada as far south as the Mediterranean in Europe and the Central Rocky Mountains in North America (Thomas et al. 2007). Across this range, juniper displays striking phenotypic variability that has been hypothesized to be a result of both phenotypic plasticity as well as distinct genetic adaptations (Knyazeva and Hantemirova 2020; Sullivan 2001; Thomas et al. 2007; Ward 2007). For example, *J. communis* can grow as upright mid‐story trees, sprawling shrubs, or prostrate ground‐hugging stems depending on its environment (Sullivan 2001; Thomas et al. 2007). Some researchers have hypothesized that it is juniper's phenotypic plasticity that enables it to survive in such a broad suite of habitats and act as a pioneer species after natural disturbances (Knyazeva and Hantemirova 2020; Thomas et al. 2007). The degree to which the observed phenotypic variation within *J. communis* is due to environmental and/or genetic differences is unclear.

Three subspecies of the common juniper occur in the UK: *J. communis* ssp. *communis*, *J. communis ssp. nana* , and *J. communis* ssp. *hemisphaerica*. *J. communis* ssp. *communis* has the widest range of the three subspecies in the UK, and populations of ssp. *nana* and ssp. *hemisphaerica* are restricted to the west coast of Scotland and the Lizard Peninsula in Southern England, respectively. Although the genetic status of these three subspecies is unclear, the phenotypic and genetic diversity of Scottish *J. communis* and *J. communis ssp. nana* populations was investigated by Sullivan (2001). They found that *J. communis* ssp. *communis* was capable of phenotypic plasticity, with prostrate cuttings from *J. communis* ssp. *communis* plants from harsh environments growing as shrubs or upright trees in the greenhouse, whereas *J. communis ssp. nana* did not exhibit this plasticity and maintained its prostrate habit in the greenhouse. Although they did not find clear evidence of a genetic distinction between ssp. *communis* and ssp. *nana*, they did find evidence that both the growth habit of ssp. *nana* is a genetic adaptation as well as a notably plastic response of ssp. *communis* to its environment. Here, we include one population (AR) that would be considered to be ssp. *nana* based on the work of Sullivan (2001), and our work builds on this study byincluding more than twice as many populations, extending the geographic range beyond Scotland to include both the Lake District and Southern England, and by using information on the pedigrees of individuals to elucidate the degree of genetically based phenotypic variance in *J. communis* using a common garden trial.

Despite the fact that juniper's phenotypic variation suggests a highly adaptive and resilient species, juniper has been in a state of decline for at least the past century (Sullivan 2003; Verheyen et al. 2009). Juniper occurs across the UK from Cornwall to Shetland but has substantial populations in three main geographic regions: Southern England, the Lake District, and Scotland. Southern English populations are particularly small and fragmented, whereas those in the Lake District and Scotland tend to be larger. Many populations across the UK are failing to regenerate (Sullivan 2001; Thomas et al. 2007). Understanding how genetic diversity is partitioned within UK juniper is especially important as a basis for dynamic conservation and to anticipate and mitigate the impacts of habitat fragmentation and reproductive senescence.

Here, we quantified patterns of within and between‐family variation in phenotypic traits of *J. communis* from across Britain using a common garden trial. We also compared variation between the three main British distribution centres, or regions, which were identified as genetic units in a parallel analysis of the population genetics of common junipers in Britain (Baker et al. 2024). Finally, we compared the phenotypic data from our common garden trial with climate data from source locations. The main goals of this work were:

1. to estimate the extent and pattern of quantitative genetic trait variation, particularly how variation is partitioned between regions, populations, and families;
2. to identify the primary traits showing genetic variation, suggesting that they may be important for local adaptation;
3. to identify the key abiotic factors driving local adaptation by correlating quantitative trait variation with climate variables from the source location.

## 2 Methods

### 2.1 Seed Collection

**FIGURE 1:**
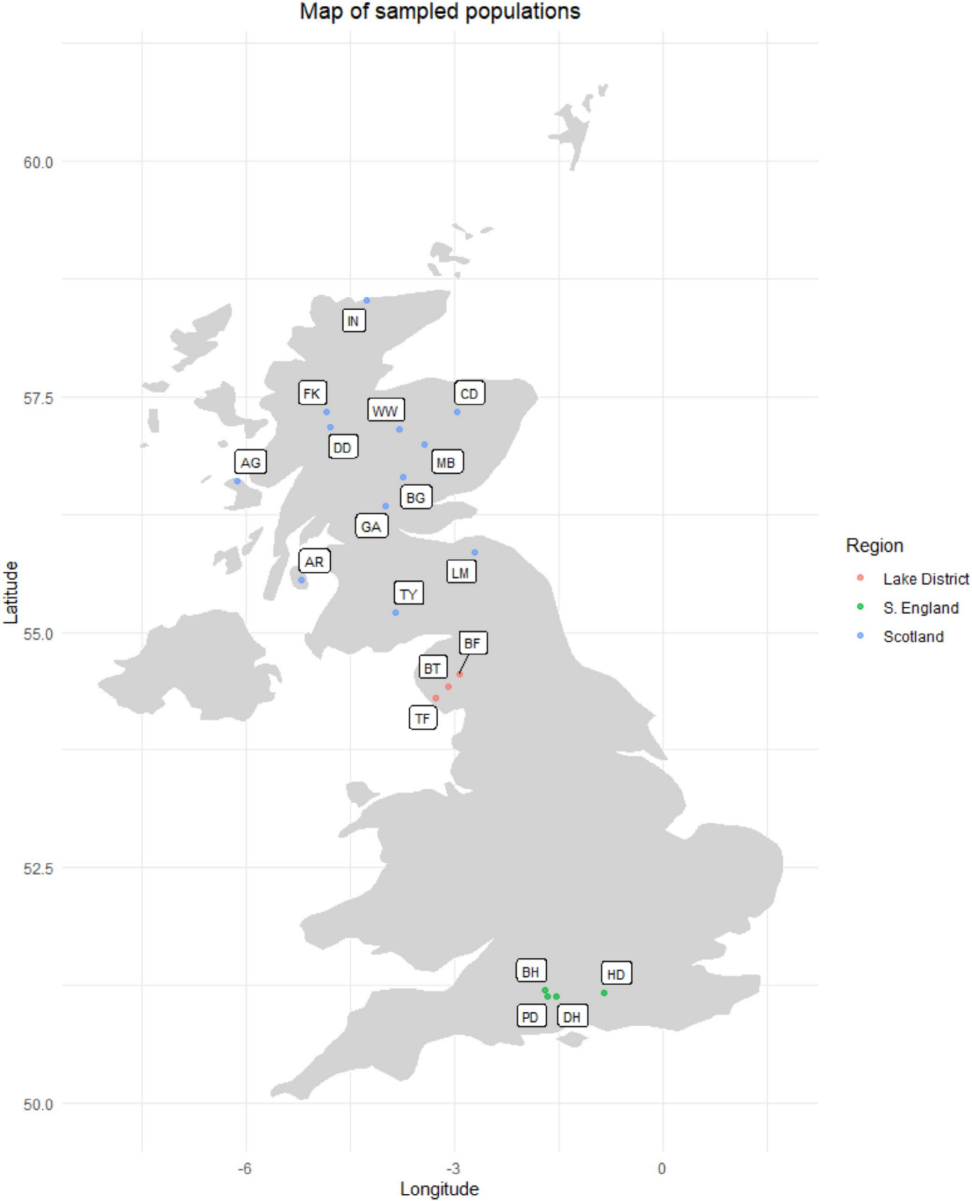
Map displaying locations of sites where trees were sampled for the common garden trial. Colors denote: Red‐Lake District, green‐S. England, blue‐Scotland. Abbreviations match those used in Table 1. Note that not all populations were included in the common garden due to low germination rates and high mortality rates of seedlings.

Seeds were collected in autumn 2015 from natural populations in England and Scotland (Table 1, Figure 1). Although the UK distribution of juniper includes populations in Wales and Ireland, no samples from these areas were included in this study. Both population and family identities were recorded for each seedlot. Population refers to the stand of juniper from which the berries were collected, and family refers to a collection of open‐pollinated seeds from one maternal tree, most likely half‐siblings. Berries were collected from a minimum of four and an average of eight maternal trees from each population, at a minimum of 25 m spacing, avoiding adjacent trees and at wider spacing where the stand size allowed. Berries were stored at 4°C until they were ready to be processed, and seeds were extracted from berries by hand. Seed viability was evaluated using the float test, where seeds that sank in water were considered viable and those that floated were assumed to be empty and therefore nonviable. A total of 26,585 viable seeds were sown (full data are available from the EIDC archive (Baker et al. 2024a)).

**TABLE 1:** List of populations collected for inclusion in the greenhouse trial with abbreviations, region assignment, latitude and longitude of the stand, the number of families from which seeds were collected, and the number of individuals that were ultimately included in the common garden trial.

Population	Abbreviation	Region	Latitude (°)	Longitude (°)	Number of families	Number of individuals
Argyll	AG	Scotland	56.605	−6.123	7	20
Gleann Dubh, Arran	AR	Scotland	55.550	−5.202	7	21
Blowick Fell	BF	Lake District	54.558	−2.931	9	30
Balnaguard Glen	BG	Scotland	56.644	−3.730	8	27
Bulford Hill	BH	S. England	51.204	−1.700	2	6
Blea Tarn	BT	Lake District	54.426	−3.088	8	24
Clashindarroch	CD	Scotland	57.337	−2.966	10	0
Dundreggan	DD	Scotland	57.185	−4.790	2	6
Danebury Hill	DH	S. England	51.137	−1.534	10	0
Fasnakyle	FK	Scotland	57.336	−4.849	1	3
Glenartney	GA	Scotland	56.340	−3.996	7	27
Harting Down	HD	S. England	51.178	−0.851	4	0
Invernaver	IN	Scotland	58.521	−4.256	3	9
Lammermuir	LM	Scotland	55.853	−2.710	9	26
Morrone Birkwood	MB	Scotland	56.998	−3.426	5	15
Porton Down	PD	S. England	51.138	−1.652	1	3
Thwaites Fell	TF	Lake District	54.302	−3.263	11	39
Tynron	TY	Scotland	55.214	−3.846	2	6
Whitewell	WW	Scotland	57.155	−3.796	7	27

### 2.2 Seed Stratification

Seeds were initially sown in seed trays (6 cm × 24 cm × 38 cm (H × W × L) with drainage holes) using a 1:1 sand: compost soil mix in December 2015. Trays were placed in loose plastic bags and subjected to a warm/cool/warm stratification protocol (MacCartan and Gosling 2013) in the greenhouse at the UKCEH, Edinburgh (latitude 55.86°, longitude −3.21°). The stratification protocol comprised mbient light at 7.3°C–14.3°C from December 2015–March 2016 followed by a cold, dark stratification period at 4°C from March 2016–October 2016, and finally, the seed trays were placed at ambient temperature and light in a greenhouse. Seedlings emerged sporadically from October 2016 to August 2018, after which the seed trays were disposed of. During four potting‐up days (October 2016, February 2017, October 2017 and August 2018), any seedlings that had emerged were picked out and transplanted into 8 × 8 × 8 cm pots with a 1:1 sand: compost mix and placed on an irrigated bench in the greenhouse at ambient light and temperature. Therefore, there were four discrete age classes, hereafter denoted 1–4, with one being the first (oldest) and four being the last (youngest) plants to emerge. These age classes were recorded for each individual to allow for the variation in traits caused by plant age to be accounted for in subsequent analyses. Each plant was labeled with its population, family, individual identity, and age class.

### 2.3 Common Garden Experiment

The common garden experiment consisted of 89 families from 16 English and Scottish populations in a randomized complete block design with one replicate in each of three blocks that was set up in the greenhouse. In December 2018, the 89 families that had at least three surviving individuals were re‐potted into 13 × 13 × 13 cm pots. The populations CD, DH, and HD were excluded at this point because no families from these populations had 3 surviving individuals. Up to 12 plants from each population were re‐potted. Six families whose seedlings emerged in two different potting waves were represented by three individuals in each of multiple age classes: they were BF103 (age classes 1 and 4), BG1 (age classes 2 and 4), GA123 (age classes 1, 2 and 4), TF13 (age classes 1 and 4), TF5 (age classes 1 and 4) and WW2 (age classes 1, 2 and 4). All other families were represented by one individual per block, meaning that the three blocks were each composed of 97 plants.

Traits were measured on plants from the three blocks, which had representatives from all 89 families. Two families, AG7 and LM14, were only represented by two individuals due to plants dying after being re‐potted in December 2018, but are nevertheless included in these analyses. Populations were represented by between one (FK and PD) and 11 (TF) families each based on seedling availability (Table 1). Therefore, traits were measured on 289 plants from 89 families representing 16 populations.

Eleven putatively adaptive traits that were identified by Sullivan (2001) were measured during December 2020. They are described in Table 2. Block and age class were also recorded for each plant to include as control factors in the subsequent analyses. All traits were measured in the three complete blocks with the exception of needle length and needle width, which were not recorded in block 1. Seven and nine families were removed from the analyses of needle length and width, respectively, due to only having a measurement from one individual. The removed families were: AG7, BF106, BH9, IN3, LM22, MB8, and TY6 for needle length and AG1, AG7, BF106, GA128, IN3, LM22, MB8, and TY6 for needle width.

**TABLE 2:** Summary of all 11 measured traits, including brief descriptions of how traits were measured and the measurement unit.

Trait	Description	Measurement unit
Stem length	The mean length of the main stem(s) of the plant; in the case of multi‐stemmed plants up to 4 measurements were taken on the largest stems and averaged.	cm
Stem diameter	The mean of two measurements of the diameter of the main stem(s) of the plant taken just above the soil	mm
Stem angle	The mean angle of the leading stem(s) taken with a protractor just above the soil (0°–90°) and rounded to the nearest 5° or 10°; in the case of multi‐stemmed plants up to 4 measurements were taken on the largest stems and averaged.	Degrees
Number of internodes	The number of internodes within 15 cm of the tip of the main stem	Count
Stem branching	The number of branches within 15 cm of the tip of the main stem	Count
Internode length	Mean length of 3 measurements of the internode distance of the main stems.	cm
Leaf length	The mean length of 3 randomly selected needles from where it attaches to the stem to the needle tip.	mm
Leaf width	The mean width of 3 randomly selected needles taken at the widest point.	mm
Branch number	The count of the number of branches that are > 50% the diameter of the main stem, plus the main stem	Count
Spread	The average of two measurements taken perpendicularly from one another to span the area of growth as seen from above the plant.	cm
Extension	The length of new growth on the main stem, defined as non‐woody growth	mm

### 2.4 Analyses of Trait Data

Statistical analyses were conducted using Minitab 2019 (Minitab 19 2024). Trait data were checked for outliers and skewedness; a maximum of two extreme outliers per trait were removed before running Grubbs outlier tests and Shapiro –Wilks normality tests on all traits to ensure the assumptions of the general linear models (GLMs). Branch number and spread had additional outliers that were removed, and branch number was square‐root transformed to correct for right skewness. To test for variation between and within regions and populations, general linear models were run using region as a fixed effect, population as a fixed effect nested within region, family as a random effect nested within population, age class as a fixed effect, and block as a random effect. These GLMs can be mathematically represented as:

$$Y_{\text{ijklm}}=\mu +\alpha_{i}+\beta_{j\left(i\right)}+\gamma_{i}+u_{k\left(j\right)}+v_{m}+\epsilon_{\text{ijklm}}$$

Where *μ* is the overall intercept, *α*_i_ is the fixed effect of the *i*th region, *β*_j(i)_ is the fixed effect of the *j*th population nested within the *i*th region, γ_l_ is the fixed effect of the *l*th age class, *u*_k(j)_ ~ *N*(0,σ^2^_u_) is the random effect of the *k*th family nested within the *j*th population, *v*_m_ ~ *N*(0,σ^2^_v_) is the random effect of the *m*th block, and ϵ_ijklm_ is the residual error term. To test for variation within age classes, GLMs were run within the youngest and oldest age classes using the same factors and nesting as above, excluding age class. A Bonferroni correction for multiple tests was applied to all general linear models, giving a revised significance level of *p* = 0.0045. The results of these models can be found in Appendix 1.

**FIGURE 2:**
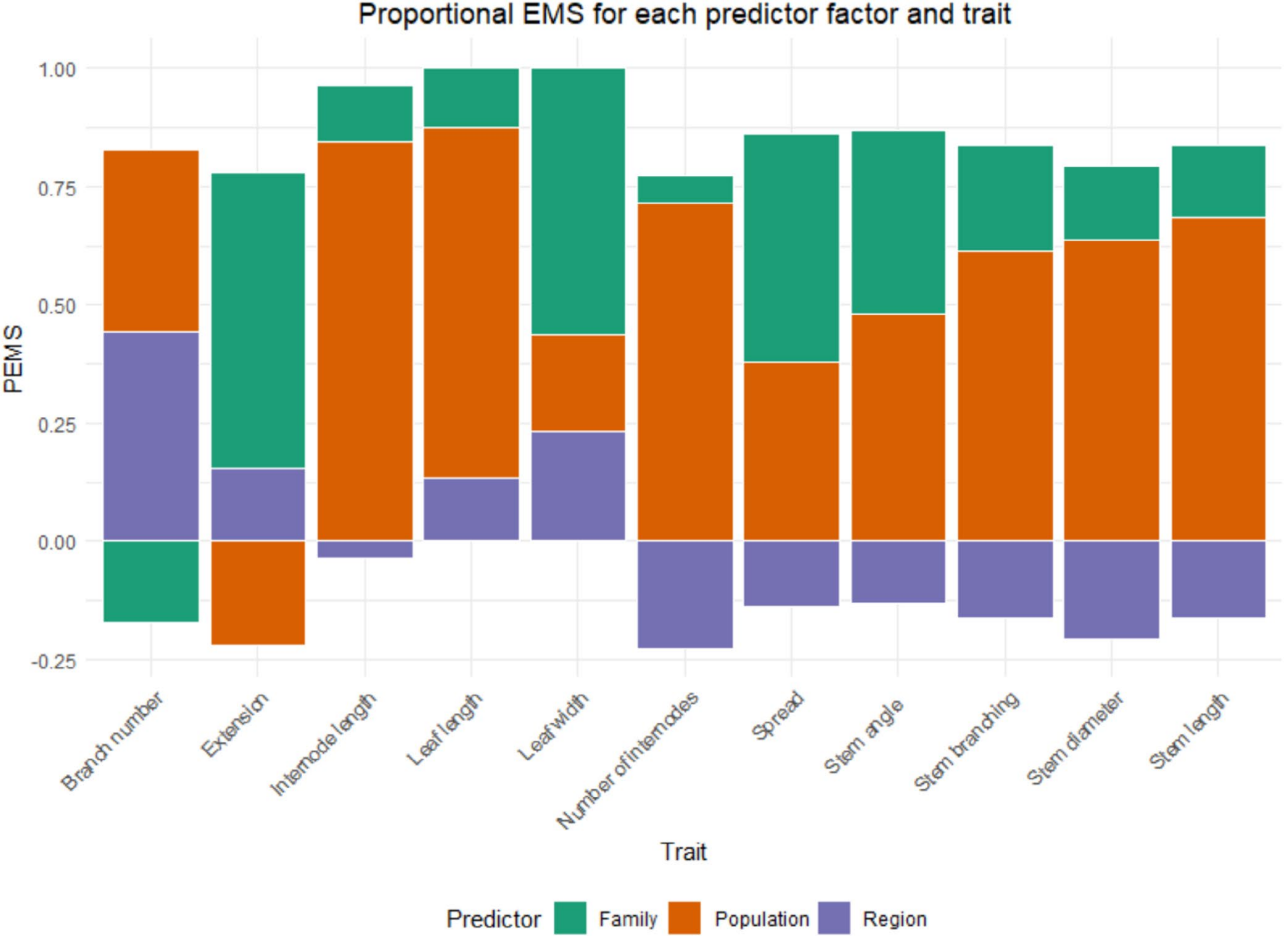
Proportional Expected Means Squared (PEMS) values for region, population, and family for each trait, presented as percentages of the sum of expected means squared values. Represents the proportion of the observed variation that each predictor factor was ascribed to by general linear models. A negative PEMS value indicates that the inclusion of the predictor did not improve the model. General linear models were calculated with region (fixed factor), population (fixed factor) nested within regions, family (random factor) nested within populations, and both age class (fixed factor), and block as a (random factor). PEMS values were calculated only from means squared values from predictors of interest.

Proportional Expected Means Squared (PEMS) values were calculated for each predictor except the control factors (block and age class) from Adjusted Means Squared (AMS) values by subtracting the AMS of each predictor from the level of nesting “below” it and dividing that by the average number of individuals in that nesting level. For example, PEMS of families is equal to the AMS for families, minus the AMS for the error term divided by the average number of individuals per family. The sum of these values was used to calculate the PEMS for each predictor (Figure 2).

Narrow‐sense heritability (*h* ^*2*^) was calculated by multiplying the EMS of family by four to account for average sibling relatedness and dividing by the sum of EMS values for all predictors and the error term. For example, the *h* ^*2*^ of stem length is equal to (EMS Family*4)/Sum EMS = (9.7*4)/145.9 = 0.265.

### 2.5 Accessing and Processing Climate Data

Correlations between trait measurements and climate variables were conducted only for Scottish sites as this removed the potentially confounding effect of regional population genetic structure (Baker et al. 2024) while maintaining sample size. Data on climate variables for the 30‐year period 1989–2019 were gathered from HadUK‐Grid (Hollis et al. 2018) on a 1km^2^ resolution for each of the 9 Scottish sites. We included: average February minimum temperature, average annual cumulative rainfall, average annual temperature, and average annual cumulative days with ground frost. Monthly means for each variable were converted from .nc to .csv files using the “raster” package for R (Hijmans 2023), then processed into annual means as needed on base R (Team 2021). Processed values that were used in the preceding Principal Component Regressions (PCR) for each site can be found in Appendix 2.

### 2.6 Regression of Trait Data on Climate Variables

Principal Component Regressions (PCRs) were used to evaluate the influence of climate at the site origin on trait variations. PCR is capable of handling multicollinearity among predictor variables (Kovoor and Nandagiri 2007) and is commonly used to simplify complex climatic datasets and identify the most important predictors (Marenco and Antezana‐Vera 2021). Minitab 19 (Minitab 19 2024) was used to run a Principal Component Analysis (PCA) on the four climate variables. The first two principal components were used as predictor variables in regression analyses with each of the 11 traits. Since climate data were on a 1km^2^ resolution, which typically encompasses the entire areas of the stands, trait data were averaged by population to compare with the climate data. To control for differences between age classes, these regressions were run within both the youngest and oldest age classes. To account for the number of tests that were performed, a Bonferroni correction was applied, giving a revised significance level of *p* = 0.0045.

## 3 Results

### 3.1 Variation of Trait Data

The main contributor to the observed variation in 6 of the 11 traits was the difference between populations (Figure 2). Although region was only a significant predictor for branch number, the inclusion of regions improved the models for leaf length, leaf width and extension. The six traits for which population was significant were: stem length (*p* = 3.70E‐07), stem diameter (*p* = 1.05E‐05), number of internodes (*p* = 1.45E‐04), stem branching (*p* = 1.67E‐05), internode length (*p* = 2.87E‐04) and leaf length (*p* = 3.90E‐05). Finally, family was significant for leaf width (*p* = 3.37E‐03) and spread (*p* = 2.76E‐03) and accounted for a large portion of the variation in both. Both age class and block were important control factors, being significant predictors for four and five traits, respectively, and so both were retained in all models (Table 3).

**TABLE 3:** Adjusted mean squared (Adjusted MS) and degrees of freedom from general linear models and calculated narrow‐sense heritability (H2) comparing 11 phenotypic traits measured from a common garden trial of 289 *J. communis* plants. Tests were nested general linear models using regions (fixed factor), populations (fixed factor) nested within regions, families (random factor) nested within populations, age class as a fixed control factor, and block as a random control factor.

		Stem length	Stem diameter	Stem angle	Number of internodes	Stem branching	Internode length	Leaf length	Leaf width	Branch number	Spread	Extension
Model adjusted R2		45.40%	45.54%	11.94%	16.87%	24.11%	24.58%	36.9%	47.5%	5.40%	44.94%	15.1%
Region	Adjusted MS	87.72	0.7927	0.4203	28.09	30.08	0.0383	21.16 **	0.1848 **	0.4921	75.48	1202.72
	DF	2	2	2	2	2	2	2	2	2	2	2
Population	Adjusted MS	742.21***	14.63**	949.34	107.77**	129.97***	25.87*	9.53**	0.0701	0.2272	231.29	873.43
	DF	11	11	11	11	11	11	11	11	11	11	11
Family	Adjusted MS	130.34	3.98	516.87	27.83	30.25	7.46	2.38	0.0403*	0.1199	91.49	872.96
	DF	72	72	72	72	72	72	65	63	72	72	64
Age class	Adjusted MS	1746.54***	49.24***	558.33	11.31	0.7566	57.55***	5.04*	0.0617	0.1035	966.68***	594.44
	DF	4	4	4	4	4	4	3	3	4	4	4
Block	Adjusted MS	395.44	20.53*	971.91	187.33*	0.4981	0.8034	3.01	0.1864*	0.3179	422.78*	5954.41*
	DF	2	2	2	2	2	2	1	1	2	2	2

*Note:* Stars next to adjusted MS values indicate significance levels: Key: *p between 0.0045 and 0.00045, **p between 0.00045 and 0.000045, ***p < 0.000045.

The narrow‐sense heritability (*h* ^*2*^) values for traits were slight to moderate, with an average value of 0.281 (Table 3). The two exceptions of leaf width (0.944) and branch number (−0.115) are likely explained by the relative PEMS values for these traits, with the variation in leaf width being predominantly ascribed to differences between families and the PEMS for family in the model for branch number being negative due to family accounting for very little variation. The narrow‐sense heritability for leaf width, which may already be quite high, would also be inflated by accounting for relatedness between individuals, whereas that for branch number is likely an error due to family not being an important predictor for this trait.

In the general linear models that were run for age classes one and four, region was a significant predictor in both age classes for leaf length (*p* = 4.8 × 10^−4^ for age class one and *p* = 2.6 × 10^−3^ for age class four). Population was a significant predictor in both age classes one and four for stem length (*p* = 3.6 × 10^−3^ for age class one and *p* = 3.9 × 10^−4^ for age class four). Within age class one, population was a significant predictor for spread (*p* = 2.0 × 10^−3^). Within age class four, population was a significant predictor for stem diameter (*p* = 7.4 × 10^−4^), number of internodes (*p* = 7.4 × 10^−4^) and internode length (*p* = 4.0 × 10^−3^). Family was a significant predictor for leaf width in age class four (*p* = 3.1 × 10^−3^). Block was a significant predictor for internode length (*p* = 3.8 × 10^−3^) in age class one (Appendix 1).

### 3.2 Correlation of Trait and Climate Variables

In the PCA of climate variables, the first two principal components (PCs) accounted for 90.1% of the observed variation (Tables 4 and 5). PC 1 was positively associated with temperature and rainfall, especially February minimum temperature, while PC 2 was positively associated with days of ground frost and average temperature.

**TABLE 4:** Eigenvalues and proportions of observed variation in the first two principal components (PC) from the PCA that was run on climate data from each of the 9 Scottish sites. Variables included in this PCA are listed in Table 5.

	PC 1	PC 2
Eigenvalue	3.0633	0.5426
Proportion variation explained	0.766	0.136
Cumulative variation explained	0.766	0.901

**TABLE 5:** The contribution of each climate variable to the first two principal components from the PCA run on climate data.

Variable	PC 1	PC 2
Average February minimum temperature	0.561	0.004
Average annual cumulative rainfall	0.489	0.029
Average annual temperature	0.476	0.684
Average annual days with ground frost	−0.469	0.729

Although only the regressions against leaf width in age class one and stem diameter in age class 4 were significant, R^2^ values were as high as 79% for some of the nonsignificant regressions. R^2^ values were equal to or larger than 50% for stem length, stem branching, internode length, and leaf width in age class one and for stem diameter, stem branching, internode length, spread, and extension in age class 4 (Table 6). For each of these traits except leaf width and stem branching, the coefficient was negative. PC 2 was not significant for any trait means.

**TABLE 6:** Results of principal component regression of all traits against climate principal components one and two (PC 1 and PC 2). Regressions were run between the average values of each trait for both age classes within each population as the response variables and climate PC 1 and PC 2 as predictor variables.

	Trait	Stem length		Stem diameter		Stem angle		Number of internodes		Stem branching		Internode length	
	Age class	1	4	1	4	1	4	1	4	1	4	1	4
Regression	R^2^	66%	38%	40%	78%	0%	6%	36%	38%	49%	59%	60%	54%
PC 1	Coefficient	−	−	−	‐ *	−	−	+	+	+	+	−	−
PC 2	Coefficient	+	−	−	+	+	−	+	+	−	−	+	−
	Trait	Leaf length		Leaf width		Branch number		Spread		Extension			
	Age class	1	4	1	4	1	4	1	4	1	4		
Regression	*R* ^2^	32%	1%	78.65% *	27%	31%	32%	45%	79%	19%	51%		
PC 1	Coefficient	−	−	+ *	+	−	−	−	−	−	−		
PC 2	Coefficient	+	−	−	+	+	+	−	+	+	−		

*Note:* Model *R* ^2^ values are displayed as percents; whether the coefficients were positive or negative is denoted with (+) and (−), respectively, and significance levels for the regression and for both PC 1 and PC 2 are displayed as stars next to the *R* ^2^ value or coefficient sign, respectively: Key: *p between 0.0045 and 0.00045.

## 4 Discussion

This paper quantifies the genetic variation in phenotypic traits of 16 natural juniper populations across juniper's three main distribution centres in Britain. Our results indicate that differences between populations explain a majority of the observed variation in half of the measured traits (6 of 11), that region is an important predictor for four traits (leaf length, leaf width, branch number and extension), and that family accounts for a large proportion of variation in four traits (stem angle, leaf width, spread and extension) and is a minor component of the observed variation in all other traits. The values of narrow‐sense heritability generally showed that the measured traits are slightly to moderately genetically based. Furthermore, our analyses indicate that weak but detectable correlations exist between trait variations and source climate variables and suggest that plants from colder, drier environments were larger than those from warmer, wetter environments when grown in the greenhouse. Finally, these correlations may be related to the age of the plants, as our comparisons between traits within the oldest and youngest age classes suggest that ontogenetic variation in resource partitioning may play a role in seedling establishment. More work is warranted to gain a full understanding of the environmental drivers of selection and how they might influence growth differently as plants age. The trial has been established outdoors in the Scottish Borders with the intention of continuing to assess these traits as the plants age.

The large differences in phenotype between populations and the similarities within them (Figure 2) indicate that juniper stands in Britain are highly adapted to their local environments (Klimko et al. 2007; Knyazeva and Hantemirova 2020; Rehfeldt 1997). The observed pattern of lower among‐family than among‐population variation is distinctive and suggests that there have been limitations to gene flow between populations to cause their phenotypic differentiation. These findings are consistent with those of both Rehfeldt (1997) and Klimko et al. (2007), who studied the quantitative genetics of seven *Cupressus* taxa and of *J. oxycedrus* with varying levels of fragmentation, respectively, using common garden trials. The results of these studies suggest that either range contractions or genetic isolation can cause relatively higher inter‐population phenotypic variation compared to intra‐population variation in two species closely related to juniper. Interestingly, Scots pine and other conifers generally do not share this pattern; in Scots pine, the majority of observed variation in needle anatomy (Donnelly et al. 2016), phenology (Salmela et al. 2013) and resistance to disease (Perry et al. 2016), among other traits, was attributed to among‐family variation, and among‐population differences were small. Researchers have hypothesized that despite fragmentation of Scots pine populations, this is due to extensive gene flow between populations that maintains variation even in the face of strong local selection (Donnelly et al. 2016; Perry et al. 2016; Salmela et al. 2013). This contrast implies a marked difference in gene flow capability between these taxa, with juniper being relatively more limited than Scots pine.

Several caveats are worth mentioning here. Firstly, since our individuals are likely half siblings but potentially full siblings, the phenotypic variation accounted for by families in this study is likely an underestimation of the true value by as much as half to three‐quarters. Furthermore, phenotypic variation is shaped by natural selection. The pattern observed in this study, of high inter‐population variation and low intra‐population variation, may be due either to stabilizing selection within stands, causing a centering of phenotypes around a “more fit” mean value, or by divergent selection, where different selective pressures among populations cause phenotypic differentiation despite gene flow between them. Either or both aspects of natural selection may be responsible for increasing inter‐population variation relative to intra‐population variation. Alternatively, the observed pattern of phenotypic variation may be due to barriers to gene flow between sites and/or a loss of genetic diversity from inbreeding, either or both of which would also cause the observed differentiations among populations and similarities within them. Although we did not genotype the same individuals present in the common garden trial, our analysis of neutral Single Nucleotide Polymorphisms (SNPs) and Simple Sequence Repeats (SSRs) data from individuals growing in 18 natural juniper populations (J. P. Baker et al. 2024) found evidence that gene flow between populations is lower than in other northern temperate tree species and that some genetic differentiation among populations is evident. Although stabilizing selection and different selective pressures among populations seem to be affecting phenotypic variation, the evidence of genetic differentiation and relatively limited gene flow from our analyses of neutral genetic markers suggests that genetic drift may also be a factor in the phenotypic differentiation of juniper stands. Our genetic analysis is also in agreement with other population genetic studies of juniper across the British Isles, which found evidence for genetic differentiation of populations and barriers to gene flow (Merwe et al. 2000; Provan et al. 2008; Reynolds 2022).

Region was a significant predictor for leaf length and width, and including regions in our models improved them for four traits: leaf length and width, number of branches, and extension. For each of these traits, region accounted for approximately 20% of the observed variation with the exception of the number of branches, in which region accounted for over 40% of the observed variation. Generally, plants from S. England had longer needles, and those from the Lake District had wider ones. Southern English plants also displayed a distinct growth habit, with each of the nine plants having one main, upright stem (Baker et al. 2024b), which is supported by the work of Ward (2007) who studied a Southern English population and found that plants grew to an average height of about 2.7 m, with no plants shorter than 1 m. In a previous field study, Knyazeva and Hantemirova (2020) found that needles were longer in southern Eurasian juniper trees and used this difference as a basis for delineating the southern populations as a separate taxon. Although the adaptive function of these observed differences in juniper's needle morphology is unclear, in other conifer species needle morphology is an adaptively important trait in controlling a plant's response to drought (Climent et al. 2024). Previous studies on several different pine species have consistently found a negative correlation between needle length and source habitat aridity (Grill et al. 2004; López et al. 2008; Ramírez‐Valiente et al. 2021; Rodriguez 2019). However, previous work on drought tolerance in junipers from across Europe found that junipers are both highly drought resistant and that populations from highly contrasting environments have largely similar hydraulic embolism resistance, suggesting that the trait is highly conserved (Unterholzner et al. 2020). By contrast, previous studies in angiosperms have found that temperature and particularly growing season length, rather than aridity, is a primary driver in the adaptation of leaf anatomy, with trees from warmer climates growing larger leaves (Esplugas 2018; Pelham et al. 1988; Wright et al. 2017). The adaptation of needle anatomy in UK juniper populations therefore seems more analogous to that of angiosperms than to other conifers. This may be because juniper in the UK is closer to the northern edge of its range than it is to its southern edge (Esplugas 2018; Thomas et al. 2007), and therefore juniper needle adaptation in the UK may be driven more by colder and wetter conditions than it is by warm and dry ones. Indeed, winter precipitation has been demonstrated to be an important factor in juniper growth rate and growth habit in Europe (Carrer et al. 2019; Hallinger et al. 2010; Pellizzari et al. 2014), and may be contributing to the phenotypic adaptations across UK populations. However, whether juniper benefits from increased snowfall through increased microbial activity and insulation (Hallinger et al. 2010; Unterholzner et al. 2022), or if snowpack negatively impacts growth through shortening the growing season (Carrer et al. 2019; Pellizzari et al. 2014) is still unclear and likely dependent on the local climate and elevation. Hallinger et al. (2010) hypothesized that these effects represent a tipping point, where some snowpack is beneficial to juniper growth, but delayed snow melting may negatively impact growth. More work is warranted on how climatic variables interact and affect the local adaptation of junipers to identify the most important factors driving adaptation in *J. communis* .

Regressions between trait variables and climate Principal Components generally lacked the statistical power to detect significant correlations. The lack of significance in these regressions is likely due to low sample sizes (*N* = 59 and 84 for age classes one and four, respectively) that were necessitated by having to control for age class, the fact that average trait values for each population were used to accommodate the 1 km^2^ resolution of the climate data, and finally statistical overfitting of the models by including PC2. Indeed, when these PCRs were run over both age classes, all the regressions with R^2^ over 50% are significant even after the Bonferroni corrections. Although the PCRs lacked the statistical power to determine significance, the coefficients for each trait in both age classes were consistent with each other. For each of stem length, stem diameter, internode length, leaf width, and spread the coefficients for PC1 between the age classes were negative and had similar values. Some trends between climate and traits were suggested in these preliminary analyses; however, they must be interpreted with caution and warrant further research. Each trait with an *R* ^2^ value over 50% except leaf width was associated with plant growth and has a negative coefficient with both age classes, indicating that plants from colder, drier environments grew larger in the greenhouse and that plants from warmer, wetter climates had wider leaves. This may be an effect of plants from more limiting environments requiring a lower temperature threshold to initiate growth (Salmela et al. 2013); however, in the most general terms, it demonstrates that climate is an important factor affecting the local adaptation of juniper trees.

Our work has shown a tentative correlation between climate and phenotype, suggesting local adaptation, but does not fully explore which climate factors may be the most important for the adaptation of juniper stands. Further research should be conducted to pinpoint the factors driving adaptation in juniper populations. The fact that juniper plants from more stressful environments grew larger in the common garden may be a result of relieving those stressors and/or it may be a genetic adaptation to allocate more energy to growth in harsher environments. Although we cannot distinguish whether the observed correlations between climate PCs and trait data were due to relieving environmental stressors, comparisons within the oldest and youngest age classes of plants in our study hint that ontogenetic adaptation may be a factor.

The general linear models run within the oldest and youngest age classes found that populations were significantly different in multiple traits associated with growth and size within the youngest age class (stem length, stem diameter, number of internodes and internode length; *p* < 0.0045), but not the oldest (Appendix 1). These analyses suggest that plants were more variable in size and growth rate when they were younger, and that these differences became less pronounced as the plants aged, when they might have adopted more distinct growth habits and sizes. In the oldest age class, only stem length and spread were significantly different between populations (*p* < 0.0045), which may be explained by the different growth habits and sizes that the trees developed as the aged. This conclusion is consistent with other studies that hypothesize the evolution of a “go for broke” strategy where younger plants invest more in photosynthesis and transpiration to aid establishment at the expense of a higher risk of mortality (Bond 2000; Vergeer and Kunin 2013). More specifically, previous work evaluating age‐specific growth of junipers is scarce. Kramer (1989) reported that juvenile *J. occidentalis* trees from Oregon invested more in their root systems than older individuals, and older individuals, by contrast, partitioned more of their proportional growth to their foliage. Although our study did not evaluate root growth, Kramer's work does illustrate ontogenetic adaptation in a closely related species. Seedling life stages are likely subject to very strong selective pressure, as evidenced by the heavily skewed age distributions of natural stands towards older individuals and the reproductive failure of natural populations (Thomas et al. 2007). These differences between age classes in our data hint at the possibility that energy partitioning to growth may be both adaptive and age‐specific. More detailed work is needed to understand the ecological trade‐offs that may influence this putative adaptive life history pattern.

### 4.1 Conservation Recommendations

Conservation managers face a difficult situation in protecting the remnant juniper stands in the UK, particularly with the introduction of the novel pathogen *Phytophthora austrocedri* (Green et al. 2015). Although there is some evidence for natural resistance to *P. austrocedri* among UK juniper populations (Green et al. 2020), their capacity to develop this resistance may be hampered by the lack of or low levels of natural regeneration and genetic isolation. Furthermore, planting operations may play a role in the spread of *P. austrocedri* to populations within 2 km of each other (Donald et al. 2021) or even inadvertently damage the established juniper trees (De De Frenne et al. 2020). Planting into existing sites is therefore strongly discouraged. Encouraging natural regeneration in established stands, for example, using fencing to exclude grazers or sod cutting to create disturbance (De De Frenne et al. 2020; Verheyen et al. 2005) is likely the best way to facilitate the adaptive potential of juniper while mitigating the risk of *P. austrocedri*.

Although the pollen and seed dispersal of juniper is not fully understood, our work has demonstrated that juniper probably has less effective gene flow than other conifer species, which is evident in the lower levels of within‐population variation compared to between‐population variation, and which is also supported by our parallel work on neutral genetic markers (J. P. Baker et al. 2024). Therefore, the establishment of “satellite” populations—small, planted stands interspersed among remnant fragments at a minimum distance of 1 km from existing sites—may facilitate gene flow, reduce the effects of inbreeding, and help to maintain the adaptive potential of populations. This paper has also illustrated the importance of local adaptations to junipers, evidenced by the differences in adaptive phenotypes among populations, which have been selected for by their respective environments. In light of this, potential satellite populations should be composed of locally sourced material to reduce the chances of environmental mismatch. Stock for satellite populations should also be raised from seeds, rather than cuttings, to reduce the effects of inbreeding. Future work should focus on juniper's gene dispersal capacity and reconnecting population fragments. Although the guidance to source & grow locally may appear to contradict a call to increase connectivity, we recommend that wherever possible natural gene flow connections are encouraged, rather than transplantations, to minimize the biosecurity risks associated with moving plant materials. Finally, this paper shows the distinct phenotypic differentiation of juniper populations across Britain. The authors recommend that several of the populations across the range studied here be considered for designation as Gene Conservation Units under the EUFORGEN framework (Hubert and Cottrell 2014) based on both the findings presented here and in our companion paper on neutral genetic markers (J. P. Baker et al. 2024).

## Author Contributions

**J. Baker:** data curation (lead), visualization (lead), writing – original draft (lead), writing – review and editing (equal). **J. Cottrell:** conceptualization (equal), project administration (equal), supervision (equal), writing – review and editing (equal). **R. Ennos:** conceptualization (equal), project administration (equal), supervision (equal), writing – review and editing (equal). **A. Perry:** conceptualization (equal), project administration (equal), writing – review and editing (equal). **S. Green:** methodology (lead), writing – review and editing (equal). **S. Cavers:** conceptualization (lead), project administration (lead), supervision (lead), writing – review and editing (equal).

## Conflicts of Interest

The authors declare no conflicts of interest.

## Data Availability

The data described in this article are accessible open‐access through the Environmental Information Data Centre (EIDC). Please see Baker et al. (2024b) (DOI: https://doi.org/10.5285/330cf3ac‐21c3‐4fa8‐ab76‐528f8cb2fbb8) for the trait data described here, and (Baker et al. 2024a) (DOI: https://doi.org/10.5285/f61dcdcc‐b838‐4bfa‐8d94‐7820114a68c8) for the germination and seedlot collection data for the full trial.

## Appendix 1

Results from general linear models run for all traits within the oldest (1) and youngest (4) classes. Tests were nested general linear models using regions (fixed factor), populations (fixed factor) nested within regions, families (random factor) nested within populations and block as a random control factor. Stars next to adjusted MS values indicate significance: Key: **p* between 0.0045 and 0.00045, ***p* between 0.00045 and 0.000045.

**Table:**

	Trait	Stem length		Stem diameter		Stem angle		Number of internodes		Stem branching		Internode length	
	Age class	1	4	1	4	1	4	1	4	1	4	1	4
	Model Adjusted R^2^	31.4%	30.6%	19.9%	20.2%	7.6%	10.3%	0.0%	26.5%	25.9%	9.1%	33.8%	16.6%
Region	Adjusted MS	101.37	123.4	0.545	0.486	612.9	795.8	10.97	17.62	31.207	43.641	6.295	19.23
	DF	2	2	2	2	2	2	2	2	2	2	2	2
Population	Adjusted MS	355 *	604.8 **	8.03	13.1738 *	796.1	610.1	23.84	112.22 *	56.742	47.606	10.781	26.933 *
	DF	12	9	13	9	12	9	12	9	12	9	12	9
Family	Adjusted MS	122.17	143.9	3.759	3.327	415.3	540.7	22.42	28.83	27.578	25.559	10.657	8.432
	DF	41	40	41	40	41	40	41	40	41	40	41	40
Block	Adjusted MS	81.17	133.5	4.743	6.256	251.1	497.3	67.79	62.69	8.525	1.546	34.843 *	1.804
	DF	2	2	2	2	2	2	2	2	2	2	2	2
	Trait	Leaf length		Leaf width		Branch number		Spread		Extension			
	Age class	1	4	1	4	1	4	1	4	1	4		
	Model Adjusted R^2^	47.1%	35.2%	50.0%	53.7%	21.8%	2.6%	32.4%	18.3%	12.0%	27.3%		
Region	Adjusted MS	20.071 *	16.1453 *	0.206	0.247	0.447	0.117	59.54	174.16	375.9	228.9		
	DF	2	2	2	2	2	2	2	2	2	2		
Population	Adjusted MS	4.792	6.0873	0.0440	0.0322	0.242	0.253	215.76 *	131.08	588.5	769		
	DF	12	9	12	9	12	9	12	9	12	9		
Family	Adjusted MS	2.34	2.5361	0.0338	0.0507 *	0.132	0.093	67.25	67.68	828	927.7		
	DF	33	37	33	37	41	40	41	40	38	39		
Block	Adjusted MS	2.658	0.0025	0.0912	0.00403	0.217	0.224	152.56	179.24	3495	1821.6		
	DF	1	1	1	1	2	2	2	2	2	2		

## Appendix 2

Forty‐year averages of climate variables for each Scottish site that were subsequently used in the PCR. The average and standard deviation (Stdev) of all populations are included at the bottom of the table.

**Table:**

Pop	Febuary min. temp (°C)	Annual cumulative rainfall (mm)	Average annual temp (°C)	Annual cumulative days with groundfrost (days)
AG	1.93	2017.36	8.62	82.46
AR	1.71	2126.64	8.69	111.97
BG	0.30	956.14	8.59	138.50
GA	0.49	1407.27	8.68	132.54
IN	1.02	1113.89	8.13	89.06
LM	0.10	1051.33	7.32	124.54
MB	−1.53	1059.20	6.84	144.73
TY	0.84	1394.97	8.30	118.63
WW	−0.55	880.66	7.46	139.53
Average	0.72	1473.49	8.46	116.86
Stdev	0.9486	658.76	0.9959962	20.206172
